# Graves’ disease in Children: A Case Report of Rare Occurrence

**DOI:** 10.12669/pjms.41.4.10689

**Published:** 2025-04

**Authors:** Afia Tariq Butt, Ayaz Ur Rehman, Sobia Ramzan, Muzna Arif

**Affiliations:** 1 Afia Tariq Butt, FCPS, Department of Pediatric Medicine and Child Health, Aga Khan University Hospital, Karachi, Pakistan; 2 Ayaz Ur Rehman, MBBS, Department of Pediatric Medicine and Child Health, Aga Khan University Hospital, Karachi, Pakistan; 3 Sobia Ramzan, FCPS, Department of Pediatric Medicine and Child Health, Aga Khan University Hospital, Karachi, Pakistan; 4 Muzna Arif, FCPS, Department of Pediatric Medicine and Child Health, Aga Khan University Hospital, Karachi, Pakistan

**Keywords:** Graves’s disease, Goitre and young age, Hyperthyroidism

## Abstract

Graves’ disease (GD) is an autoimmune disorder that manifests as goiter, weight loss, heat intolerance, and palpitations. It is rare in preschool-aged children (<5 years) and can lead to serious acute and long-term complications, including growth and development, if left undiagnosed.

We present the case of a four-year-old Hindu Asian girl from a low socioeconomic background who presented with progressive thyroid enlargement and symptoms of excessive sweating, heat intolerance, weight loss, diarrhea, fatigue, and palpitations over six months. Examination revealed symmetrical goiter, signs of thyrotoxicosis, bilateral exophthalmos, and raised blood pressure. Laboratory investigations confirmed Graves’ disease with suppressed TSH, elevated T3 and free T4, and persistently high TSH receptor antibodies (TRAb >40 IU/L). She was started on carbimazole and propranolol, resulting in clinical improvement; however, biochemical parameters, including TRAb levels, remain persistently elevated at eight months and suppressed TSH at 13 months, indicating a high risk of relapse. Long-term ATD therapy will be continued, with definitive treatment (RAI or thyroidectomy) considered if remission is not achieved after three years. This case underscores the challenges of managing Graves’ disease in young children and the need for prolonged monitoring and individualized treatment strategies.

## INTRODUCTION

Hyperthyroidism is a pathological state characterized by increased synthesis and secretion of thyroid hormones thyroxine (T4) and triiodothyronine (T3) by the thyroid gland. Hyperthyroidism is uncommon in preschool-aged children.

In northern Europe, the incidence of thyrotoxicosis in young children is 0.1 per 100,000 person-years^1^ unlike adults; children rarely experience severe ophthalmopathy and dermatologic symptoms. Common causes of hyperthyroidism in children include Graves’ disease, toxic multinodular goiter, thyroiditis (including Hashimoto’s thyroiditis in its early stages), and thyroid adenomas. Graves’ disease is an autoimmune thyroid disorder characterized by the production of autoantibodies targeting the thyroid-stimulating hormone (TSH) receptor. These antibodies stimulate the thyroid gland, resulting in excessive synthesis and release of thyroid hormones, specifically thyroxine (T4) and triiodothyronine (T3). The most common sign of Graves’ disease in children is goiter followed by exophthalmos.^2^ Hyperthyroidism in children presents with symptoms like palpitations, tremors, irritability, and growth failure, with Graves’ orbitopathy rarely causing exophthalmos. Diagnosis is based on suppressed TSH, elevated T3/T4, TRAb positivity, and excluding other causes, while treatment includes anti-thyroid drugs (ATD), radioactive iodine (RAI), or surgery. Delayed diagnosis of Graves’ disease can lead to life-threatening complications such as cardiovascular abnormalities. Therefore, prompt diagnosis, management, and monitoring are crucial. Here we share our experience of Grave’s disease in a pre-school age girl treated successfully with anti-thyroid drug.

## CASE PRESENTATION

We present the case of a four years old Hindu Asian girl who was brought to our attention at outpatient department, with six-month history of progressive swelling in front of neck, associated with excessive sweating, heat intolerance, and weight loss. She also had on-and-off complaints of diarrhea, increased tiredness, and palpitations over the past six months. Her past medical history, immunization status and drug history were unremarkable. She was born at term to non-consanguineous parents, with no natal or post-natal complications. There was no family history of thyroid or other autoimmune diseases. On examination, her height was 104 cm (75th percentile) and weight was 13.5 kg (10th percentile). She appeared anxious with tremors of the arms and hands and had obvious bilateral exophthalmos and goiter. Her vitals were heart rate of 160 beats per minute, warm to the touch (temperature of 37.5°C), blood pressure was106/68 mmHg (90th percentile for height), respiratory rate of 30 breaths per minute, and 100% oxygen saturation on room air. The patient exhibited a diffusely enlarged thyroid gland (WHO grade 2) with no asymmetry, tenderness, or palpable nodules. The upward movement of thyroid during swallowing and Graefe’s sign was present but there was no audible bruit.

Laboratory investigations for hyperthyroidism were positive as evidence by low thyroid-stimulating hormone level (TSH) <0.005 μIU/mL (0.7-6.4), high triiodothyronine (T3) 9.88 nmol/L (1.62-4.14), and free thyroxin (free T4) 7.7 ng/dL (0.92-1.99). To confirm the underlying cause, autoimmune work up was done and all including thyroglobulin antibodies 14.5 IU/mL (115), thyroid peroxidase (TPO) antibodies 310 IU/mL (<34) and TSH receptor antibodies (TRAb) were >40 IU/mL (0-1.75) were positive (Table-I). Her CBC showed hemoglobin concentration 7.1 g/dL, a hematocrit of 29.2% and normal WBC count. Workup for other autoimmune disease such as celiac and Cushing disease were negative. The ultrasound revealed a prominent thyroid gland with normal echogenicity, and no nodules or abnormal vascularity. No cervical lymphadenopathy was observed. The right and left lobe measurement were 39.7 × 16.8 × 16.3 mm, and 37.0 × 18.6 × 20.2 mm respectively.

**Table-I:** Sequential Timeline of Clinical Course and Management

Parameter	Initial Visit	2-Month Follow –Up	6-Month Follow-Up	8-Month Follow-Up	13-month follow-up
Clinical					
Anthropometry	Weight: 13.5 kg (10^th^ percentile), Height: 104 cm (75^th^ percentile)	+1.5 kg weight gain	Weight: 16 kg (25^th^ percentile), Height: 109 cm (>75th percentile)	Weight: 16.5 kg (25^th^ percentile), Height: 110cm (>75th percentile)	Weight: 17kg (25^th^ percentile), Height: 116 cm (>95th percentile)
Blood pressure (BP) and Heart Rate(HR)	BP: 106/68 mmHg (90th percentile), HR: 160 bpm (tachycardia)	BP normalizing, HR improved	HR normal, BP normal	HR normal, BP normal	BP: 110/64 mmHg (90th percentile), HR: 119bpm (tachycardia)
Eye Findings	Bilateral exophthalmos	Mild improvement	Further reduction in prominence	Exophthalmos unchanged	Eye exam Normal
Other Symptoms	Sweating, diarrhea, irritability, tremors	Improved	Resolved	Fever and loose stool	Palpitation and heat intolerance
Laboratory					
Hematology	Hemoglobin: 7.1 g/dL, WBC normal	-	-	Hemoglobin: 10.8 g/dL, WBC normal	-
Thyroid Function	TSH <0.005 μIU/mL (0.7-6.4), FT4 >7.7 ng/dL (0.92-1.99) T3 9.88 nmol/L (1.62-4.14)	TSH <0.005 FT4: 3.4 ng/dL,	TSH <0.005 FT4: 1.47 ng/dL,	TSH <0.005 FT4: 1.43 ng/dL,	TSH <0.005 FT4: 1.41
Autoimmune Workup	TRAb >40 IU/L (0-1.75), TPO Antibody 310 IU/m (<34), Anti-Thyroglobulin 14.5 IU/mL (115)	TRAb still high	TRAb persistently high	TRAb >40 IU/L	_
Treatment	Carbimazole 0.8 mg/kg/day, Propranolol 5 mg BID	Carbimazole increased to 7.5 mg AM, 5 mg PM	Same doses continued	Same doses continued	Beta blocker restarted. Carbimazole increased to 7.5 mg AM, 5 mg PM

WBC, white blood cell: T3, triiodothyronine: FT4, free thyroxine: TSH, thyroid-stimulating hormone: TRAB, TSH receptor antibodies: TPO, Thyroid peroxidase antibody.

Based on clinical findings, evidence of thyrotoxicosis and autoimmune work up as well symmetrically diffuse enlargement of thyroid on ultrasound, we diagnosed her as Graves’ disease. Hyperthyroidism in Hashimoto’s thyroiditis, known as Hashitoxicosis, is typically transient and caused by the release of preformed thyroid hormones due to glandular destruction, unlike Graves’ disease, which involves sustained hormone overproduction driven by TSH receptor antibodies. Elevated TRAb and clinical persistence of hyperthyroidism in our case supported the diagnosis of Graves’ disease. She was managed with anti-thyroid drugs and B-blockers after excluding other causes and counselling of parents. Carbimazole at an initial dose of 5 mg twice a day (0.8 mg/kg/day), along with β-blockers (propranolol Inderal) at 5mg twice a day. Family was counseled regarding disease nature, compliance with anti-thyroid drug and its side effect, and proper follow up to achieve euthyroid state. The patient was initially followed every four weeks, with subsequent visits every two months once stabilized. CBC and LFT were monitored periodically to detect potential adverse effects of carbimazole, alongside clinical evaluations for side effects such as rash, joint pain, and signs of agranulocytosis or hepatotoxicity. At her two months follow-up appointment, she gained 1.5 kg weight. The dose of carbimazole was escalated to 7.5 mg in the morning and 5 mg in the evening to achieve better control of thyroid hormone levels, as her laboratory results likely indicated persistent hyperthyroidism or suboptimal improvement in free T4 and T3 levels (Table-I). At her 6-month follow-up, significant improvement was observed in anthropometric measurements (weight 16 kg, 25th percentile and height, 109 cm > 75th percentile) and clinical parameters, including normalization of heart rate, mood and behavior, decreases in exophthalmos and goiter size. Carbimazole was continued at same doses (7.5 mg in the morning and 5 mg in the evening). At eight-month follow-up, the patient has shown improvement in growth centiles and tachycardia, however FT4, TSH, TRAB levels have remained unchanged. Anti-thyroid peroxidase antibodies levels showed improvement but not normalized. On 13 months follow up she had increased palpitation with heat intolerance. Beta blocker was restarted and oral Carbimazole has been adjusted to 0.7mg/kg/dose with a follow up planned after three months with FT4, TSH and TRAB level. As the patient’s TRAb levels remain persistently high at eight months, ATD therapy will be continued, with regular TFT (thyroid function tests) and TRAb monitoring every two to three months. If TRAb remains elevated after three years of treatment, the risk of relapse will be high, and definitive treatment (RAI or thyroidectomy) will be considered. Given the patient’s young age (<5 years), RAI is not currently an option, but it may be reconsidered later. Thyroidectomy will be considered if the goiter enlarges, symptoms worsen, or there are significant adverse effects of ATD therapy.

## DISCUSSION

This case report describes Graves’ disease in a preschool-aged child presenting with symmetrical goitre and hyperthyroidism. To the best of our knowledge, no such case report has been documented from Pakistan. This case emphasizes the need for individualized treatment decisions based on biochemical response and clinical course in pediatric Graves’ disease.

In Graves’ disease, TSH receptor antibodies (TRAb) have high sensitivity (up to 97%) and specificity (99%) for diagnosis and monitoring treatment response. Persistently elevated TRAb levels, as seen in our case, suggest ongoing autoimmune activity and higher risk of relapse. Thyroid scintigraphy is not required for routine diagnosis.^3^ According to the 2016 American thyroid association guidelines, thyroid scintigraphy should be done if the symptoms of thyrotoxicosis don’t clearly indicate Graves’ disease, if thyroid nodules are present, or if the diagnosis suggests a toxic adenoma or multinodular goiter. 4

The first line treatment of Graves’ disease in children is anti-thyroid medication, with carbimazole being most used. Carbimazole is a thionamide drug that inhibits thyroid peroxidase, reducing thyroid hormone production. Common side effects include agranulocytosis and liver toxicity, requiring regular monitoring of blood counts and liver function during treatment. The initial dosage of carbimazole is 0.4 to 0.8mg/kg/day, with a maximum limit of 30mg/day.

The European Thyroid Association’s 2018 guideline suggests a 24- to 36-month course of methimazole (MMI) for pediatric patients.^5^ More recent studies and reviews advocate for even longer treatment durations, recommending at least three years and potentially extending to five years or more, especially in cases with a low likelihood of remission.^6^

Recent studies have reported a relapse rate of 57.4% after stopping ATD treatment.^7^ Key risk factors include younger age at diagnosis, larger goiter size, higher initial FT4/T3 levels, and persistently elevated TRAb levels^8^.

Indications for definitive treatment may include relapse despite extended medical treatment (at least three years of ATD), serious and persistent drug side effects, contraindications to anti-thyroid medications, poorly controlled hyperthyroidism due to non-compliance, large goiter causing compressive symptoms, requests from the family or child for personal reasons.^5^ RAI therapy is contraindicated in this patient due to her age (<5 years), as younger children have a higher risk of adverse long-term effects.

The earlier the onset of Graves’ disease, the more it is likely to have a severe disease with less chances of remission. Usual remission rate is 17.8 % after at least two years of treatment. 7 However in small children treatment of Graves’s diseases can have a very difficult course and most of them might require a thyroidectomy later in life due to failure to go into remission. Educating the family about the nature of the disease, the importance of medication adherence, and the need for regular follow-up visits is crucial for achieving optimal outcomes. Additionally, addressing any psychosocial aspects, such as the child’s anxiety and the family’s concerns about the disease and its treatment, is an important component of comprehensive care.^9^

## CONCLUSION

This case highlights the importance of long-term follow-up, as persistently high TRAb levels at eight months indicate a high relapse risk, requiring prolonged ATD therapy with close monitoring. Definitive treatment (RAI or thyroidectomy) will be considered if remission is not achieved after three years, or if complications arise. This case emphasizes the need for individualized treatment decisions based on biochemical response and clinical course in pediatric Graves’ disease.

### Authors’ Contribution:

**ATB:** Concept, design, interpretation and analysis of data and final revision of manuscript.

**AR:** Did data collection and manuscript writing.

**MA:** Final revision of manuscript.

**ATB** and **AR:** These are responsible and accountable for the accuracy and integrity of the work. All authors have read and approved final version of manuscript.

## Availability of data and materials:

All data generated or analyzed during this study are included in this article.
